# Effect of maturation on muscle quality of the lower limb muscles in adolescent boys

**DOI:** 10.1186/1880-6805-33-30

**Published:** 2014-09-19

**Authors:** Yuko Fukunaga, Yohei Takai, Takaya Yoshimoto, Eiji Fujita, Masayoshi Yamamoto, Hiroaki Kanehisa

**Affiliations:** 1 National Institute of Fitness and Sports in Kanoya, 1 Shiromizu, Kanoya, Kagoshima 8912393, Japan

**Keywords:** Puberty, Maximal voluntary contraction, Muscle volume, Pubic hair, TQ-MV relationship

## Abstract

**Background:**

The purpose of this study was to clarify the effect of maturation on the muscle quality of the lower limb muscles around puberty.

**Methods:**

Subjects were 117 Japanese boys age 12 to 15 years. The maturity status was assessed by using a self-assessment of stage of pubic hair development based on the criteria of Tanner. On the basis of the criteria, subjects were divided into the prepubescent or pubescent group. Muscle thickness of knee extensors and plantar flexors were measured by a B-mode ultrasound. Muscle volume index (MV) was calculated from muscle thickness and limb length. Maximal voluntary isometric joint toques (TQ) of knee extension and ankle plantar flexion were measured using a myometer. Muscle quality was derived from dividing TQ by MV (TQ/MV).

**Results:**

In both muscles, TQ-MV relationships were also similar between the prepubescent and pubescent groups, and there was no significant difference in TQ/MV between the two groups when chronological age was statistically adjusted.

**Conclusion:**

The current results indicate that, for adolescent boys, the muscle quality of the lower limb muscles is not significantly influenced by maturation.

## Introduction

It is well-known that, in adults, muscle size is a major determinant of force production capacity [1-6]. On the other hand, the earlier findings on the relationship between force-production capacity and muscle size during growth period differ among studies and among muscle groups tested [7-13]. One reason is that growth of both strength and muscle size is affected by chronological age and maturation [14]. Furthermore, methodological difference in determining muscle size [15] may also be a reason for the aforementioned discrepancy among the previous findings on strength-size relationships. The specific tension of a muscle is theoretically determined as muscle force relative to the physiological cross-sectional area (PCSA). To determine precisely the PCSA of a muscle *in vivo* is difficult because of the need to measure muscle volume, muscle fiber length, and fiber pennation angle [16], but it is difficult to measure these precisely *in vivo*. Some researcher have used the maximal voluntary joint toque (TQ) per muscle volume (TQ/MV) as an index of muscle force per PCSA [6], and this index is expressed as muscle quality [17,18]. Furthermore, Akagi *et al*. [4] have demonstrated that MV compared to muscle anatomical cross-sectional area is appropriate for evaluating the strength-size relationship in elbow flexors. These aspects indicate that maturity-related difference in strength-size relationships should be examined by using the TQ-MV relationship.

Some studies have already examined the TQ-MV relationship and muscle quality in periods of growth [12,13,19]. However, the issue concerning the influence of maturation on muscle quality is still controversial. Pitcher *et al*. [19] demonstrated that TQ/MV in the knee extensors was constant for 6 months in the period of preadolescence. On the other hand, the specific force of the gastrocnemius muscle is higher in early prepubescent boys than in adults [13]. Knee extensor muscle strength in boys is influenced not only by body size but also by testosterone level [20], which becomes an indicator of maturation. Serum testosterone level is positively related to maximal isokinetic knee extension in adolescent boys [21]. Taken together, muscle quality of the lower extremity muscles would be influenced by maturation.

The earlier findings cited above have been obtained by comparing prepubescent boys with adults. During adolescence, body size markedly changes with advancing chronological age and maturation, and its change accompanies with increases in muscle size and strength [22,23]. It has been shown that not only chronological age but also the magnitude of maturity influences the development of qualitative factors such as muscle strength, fiber composition, glycolytic and motor coordination [20,21,24]. This may complicate the interpretation of TQ-MV relationship in growth period. It should be necessary to determine the effect of maturation on muscle quality within a limited chronological age range in order to reduce the influence of chronological age on maturity-related difference [25,26]. To the best of our knowledge, less information on the influence of maturation on the TQ-MV relationship around puberty is available from earlier studies. Force production capacity of the lower extremity muscles (for example, the knee extensors and ankle plantar flexors) is associated with sprint and jump performances [12,26,27]. To clarify the influence of maturation on muscle quality is feasible for coaches and physical educators involved in pediatric exercise. Hence, the purpose of this study is to clarify the effect of maturation on muscle quality in the lower extremity muscles around puberty for adolescent boys. Considering the earlier findings cited above, we hypothesized that the TQ-MV relationship in the lower limb muscles would differ between prepubescent and pubescent boys, and the muscle quality might be higher in pubescent than in prepubescent boys.

## Methods

### Subjects

One hundred and seventeen boys age 12 to 15 years participated in this study. Prior to the experiment, this study was approved by the Ethical Committee of the National Institute of Fitness and Sports in Kanoya and was consistent with their requirements for human experimentation. All subjects and their parents were informed of the purpose and procedures of this study and possible risks of the measurements beforehand. Written informed consent was obtained from each subject and parent.

### Experimental design

Malina *et al*. [14] reported that Tanner stage I and II indicates criteria of preadolescence period. Age at peak height velocity, an index of puberty onset, is 13 years for Japanese boys [28-30]. We have also demonstrated that body height at peak height velocity in Japanese boys (approximately 154 cm) corresponded to that between Tanner stage II and III [26]. On the basis of the maturity status, therefore, the subjects were allocated to the prepubescent group (n = 47, Tanner stage I to II) or the pubescent group (n = 70, Tanner stage III to V). All procedures were conducted according to our previous studies [25,26,31].

### Assessment of sexual maturation

A self-assessment of stage of pubic hair (PH) based on the criteria of Tanner [32], which was illustrated with black and white, was used to evaluate secondary sex characteristics. To reduce embarrassment, each subject went into a room by himself to complete the self-assessment anonymously [33]. Once completed, the self-assessment form was put into a box set in the room. The stage of PH consisted of five classes.

### Measurements of muscle thickness

Muscle thickness of knee extensors (KE) and ankle plantar flexors (PF) were measured with a B-mode ultrasonographic apparatus (Prosound2, Aloka, Tokyo, Japan) with a linear scanner. As described in the earlier study [34], the ultrasonographic images were obtained at 50% of femur length (the distance from the greater trochanter of the femur to the articular cleft between the femur and the tibial condyles) and the proximal 30% of lower leg length (the distance from the tibial condyles and lateral malleolus). The muscle thickness (MT) was defined as the distance from adipose tissue-muscle and bone interface. The muscle volume indices (MV) of the knee extensors and ankle plantar flexors were calculated using the prediction equations derived from MT and limb length (L) reported by Miyatani *et al*. [35]:

$$\begin{array}{l} \mathrm{MV}\;\mathrm{in}\;\mathrm{the}\;\mathrm{knee}\;\mathrm{extensors}\;\left(cm^{3}\right)\\ \;=\left(\mathrm{MT}\left(\mathrm{cm}\right)\times 320.6\right)+\left(L\;\left(\mathrm{cm}\right)\times 110.9\right)‒4437.9 \end{array}$$

$$\begin{array}{l} \mathrm{MV}\;\mathrm{in}\;\mathrm{the}\;\mathrm{ankle}\;\mathrm{plantar}\;\mathrm{flexors}\;\left(cm^{3}\right)\\ \;=\left(\mathrm{MT}\left(\mathrm{cm}\right)\times 219.9\right)+\left(L\left(\mathrm{cm}\right)\times 31.3\right)‒1758.0, \end{array}$$

and muscle quality was expressed as TQ relative to MV (TQ/MV) [18] in each muscle.

### Measurements of maximal voluntary isometric joint torque

Maximal voluntary isometric joint torque (MVC) in knee extension and ankle plantar flexion was measured using a specially designed myometer (TAKEI, Niigata, Japan). The right leg was measured for all subjects. In the KE measurement, the subjects sat on the machine with a 90-degree angle at hip and knee joints. The subject’s hip was fixed by a non-elastic belt to prevent his hip from moving. Knee extension torque (KET) was calculated by multiplying the knee extension force by the lower leg length. In the planter flexion measurement, the subjects sat on the machine with knee extended. The ankle angle was 90 degrees. The subject’s ankle was secured by a non-elastic belt to prevent from moving. Planter flexion torque (PFT) was obtained in the myometer. The subjects gradually exerted muscle force from rest to maximum in 3 to 4 seconds and then sustained this force at the maximum for approximately 2 seconds. Subjects performed at least two MVC trials with a 2-minute rest between trials. If the difference in the MVC torque between two trials was >10%, an additional MVC trial was performed. The highest value among the trials was adopted for analysis.

### Statistical Analysis

Descriptive data are presented as means ± SDs. To test comparison between groups, an unpaired *t*-test was used. A one-way analysis of variance (ANOVA) was conducted to compare the maturity-related differences in the measured variables. An analysis of covariance (ANCOVA) was tested to assess the maturity-related difference in TQ/MV when adjusting chronological age as covariate, and a Bonferroni *post hoc* test was used for comparison between groups within same sex. Pearson’s product-moment correlation coefficient (*r*) was calculated to determine the relationship between TQ and MV in both muscles for each group. We compared the slopes and y-intercepts of regression lines from the TQ-MV relationships in both muscles between groups, and tested whether the y-intercept for each regression line differed from 0. Effect size was classified as trivial (*r* <0.1, *η*^2^ < 0.01), small (*r* = 0.1 to 0.3, *η*^2^ = 0.01 to 0.06), moderate (*r* = 0.3 to 0.5, *η*^2^ = 0.06 to 0.14), and large (r >0.5, *η*^2^ > 0.14) [36]. Statistical significance was set at *P* <0.05. All statistical procedures were conducted by using statistical software (SPSS 22.0 for windows, IBM, Japan).

## Results

**Figure 1:**
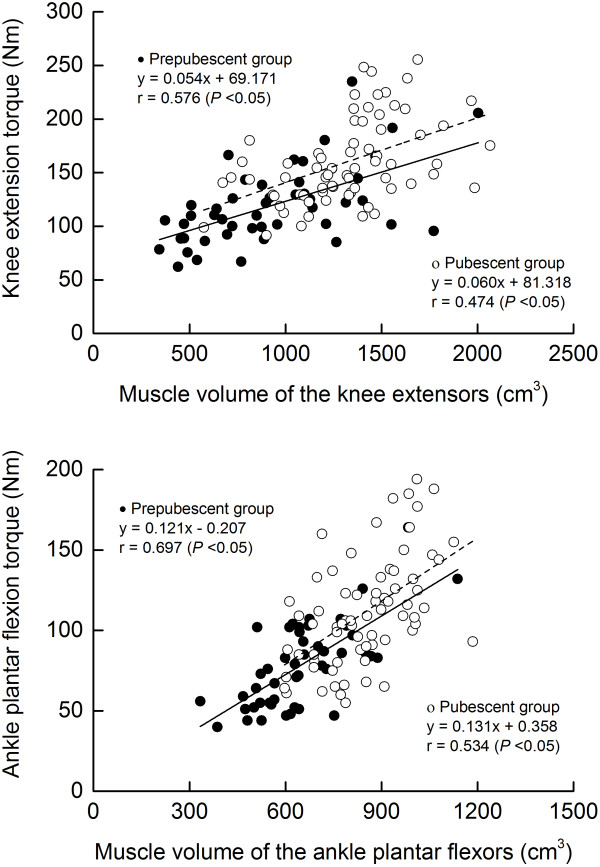
Relationship between maximal joint torque and muscle volume in the knee extensors (top) and the ankle plantar flexors (bottom) for prepubescent and pubescent boys.

The physical characteristics of the subjects are presented in Table 1. All measured variables except for KET/MV and PFT/MV were higher in the pubescent group than in the prepubescent group. TQ was significantly correlated with MV in both muscles (*r* = 0.47 to 0.70, *P* <0.05, Figure 1) with a moderate to large effect. The slopes and y-intercepts of the regression lines in the corresponding relationships did not significantly differ between the two groups. In both groups, the y-intercept of the regression line was significantly different from zero in the knee extensors, but not in the ankle plantar flexors. In the knee extensors and ankle plantar flexors, no significant difference in TQ/MV was found between the two groups when chronological age was statistically adjusted as covariate.

**Table 1:** Physical characteristics of prepubescent and pubescent boys

	**Prepubescent**	**Pubescent**	***Effect size (r)***
**Age, years**	13.3 ± 0.5	14.0 ± 0.6*	0.49
**Height, cm**	152.7 ± 8.5	163.6 ± 6.5*	0.59
**Weight, kg**	42.2 ± 8.0	51.7 ± 7.2*	0.53
**BMI, kg/m**^**2**^	18.0 ± 1.8	19.2 ± 1.5*	0.36
**Percent body fat, %**	13.6 ± 5.2	15.4 ± 3.9*	0.20
**LBM, kg**	36.2 ± 5.2	43.5 ± 4.7*	0.60
**Limb length, cm**			
**Femur**	36.7 ± 2.9	38.6 ± 2.1*	0.10
**Lower leg**	36.1 ± 2.5	38.2 ± 1.8*	0.45
**Muscle thickness, cm**			
**KE**	4.0 ± 0.5	4.6 ± 0.5*	0.49
**PF**	5.8 ± 0.5	6.4 ± 0.5*	0.53
**Muscle volume index, cm**^**3**^			
**KE**	930 ± 390	1318 ± 310*	0.51
**PF**	649 ± 152	850 ± 139*	0.55
**Maximal voluntary joint torque, Nm**			
**KET**	119.5 ± 36.7	160.0 ± 39.0*	0.47
**PFT**	78.6 ± 26.5	111.7 ± 34.2*	0.46
**TQ/MV, Nm/cm**^**3**^			
**KET/MV**	0.14 ± 0.05	0.13 ± 0.03*	0.20
**PFT/MV**	0.12 ± 0.03	0.13 ± 0.03	0.15

*indicates significant difference between prepubescent and pubescent groups.

BMI, body mass index; KET, knee extension torque; LBM, lean body mass; MV, muscle volume index; PFT, ankle plantar flexion torque; QF, thigh anterior; TQ, maximal voluntary isometric joint torque; TS, posterior of lower leg.

The physical characteristics of each PH stage are shown in Table 2. In most of the measured variables, the significant differences were observed between PH I to II and PH III to V with a moderate and large effect size. There was no significant difference in KET/MV and PFT/MV among pubertal stages. As the result of ANCOVA, in which chronological age was adjusted as covariate, no significant difference in TQ/MV was found in either muscle.

**Table 2:** Maturity-related differences in anthropometry, body composition and maximal voluntary joint torque in adolescent boys

	**PH I**	**PH II**	**PH III**	**PH IV**	**PH V**	***Significance***	***Effect size (η***^***2***^ ***)***
**Age, years**	13.0 ± 0.4	13.3 ± 0.6	13.7 ± 0.6	14.0 ± 0.6	14.1 ± 0.6	1 < 3 to 5, 2 < 4-5	0.19
**Height, cm**	146.6 ± 8.1	154.6 ± 7.7	159.7 ± 7.1	165.1 ± 5.8	165.4 ± 5.3	1 < 2 to 5, 2 < 4 to 5, 3 < 4	0.20
**Weight, kg**	38.8 ± 6.4	43.2 ± 8.2	47.4 ± 7.0	53.7 ± 5.6	53.4 ± 7.7	1 < 3 to 5, 2 < 4 to 5, 3 < 4	0.13
**BMI, kg/m**^**2**^	18.0 ± 1.6	18.0 ± 1.9	18.5 ± 1.3	19.6 ± 1.3	19.4 ± 1.7	1 < 4, 2 < 4 to 5	0.03
**Percent body fat, %**	13.8 ± 3.3	13.6 ± 5.7	14.1 ± 3.4	15.8 ± 3.9	16.3 ± 4.2		0.01
**LBM, kg**	33.3 ± 4.3	37.0 ± 5.2	40.6 ± 4.8	45.1 ± 4.0	44.4 ± 4.4	1,2 < 3 to 5, 3 < 4	0.20
**Limb length, cm**							
**Femur**	35.0 ± 2.7	37.3 ± 2.7	37.7 ± 2.1	39.3 ± 2.0	38.7 ± 2.1	1 < 3 to 5, 2 < 4	0.05
**Lower leg**	34.5 ± 2.6	36.6 ± 2.3	37.6 ± 2.0	38.5 ± 1.6	38.5 ± 1.6	1 < 2 to 5, 2 < 4-5	0.08
**Muscle thickness, cm**							
**KE**	4.1 ± 0.5	4.0 ± 0.5	4.3 ± 0.5	4.8 ± 0.4	4.6 ± 0.4	1,2 < 4 to 5, 3 < 4	0.11
**PF**	5.7 ± 0.4	5.8 ± 0.5	6.2 ± 0.5	6.6 ± 0.4	6.4 ± 0.5	1,2 < 3 to 5, 3 < 4	0.12
**Muscle volume index, cm**^**3**^							
**KE**	748 ± 380	986 ± 381	1119 ± 323	1448 ± 258	1342 ± 265	1 < 3 to 5, 2 < 4 to 5, 3 < 4	0.11
**PF**	582 ± 143	670 ± 150	787 ± 146	899 ± 115	848 ± 142	1,2 < 3 to 5, 3 < 4	0.15
**Maximal voluntary joint torque, Nm**							
**KET**	103.8 ± 29.7	124.3 ± 37.6	147.7 ± 36.3	168.0 ± 39.4	161.6 ± 39.9	1 < 3 to 5, 2 < 4-5	0.07
**PFT**	59.9 ± 16.9	84.3 ± 26.4	107.5 ± 39.3	123.4 ± 34.1	100.4 ± 23.8	1 < 2 to 5, 2 < 4	0.09
**TQ/MV, Nm/cm**^**3**^							
**KET/MV**	0.16 ± 0.06	0.14 ± 0.05	0.14 ± 0.04	0.12 ± 0.03	0.12 ± 0.03		0.01
**PFT/MV**	0.11 ± 0.03	0.13 ± 0.03	0.14 ± 0.04	0.14 ± 0.03	0.12 ± 0.03		0.01

BMI, body mass index; KE, knee extensors; KET, knee extension torque; LBM, lean body mass; MV, muscle volume index; PF, ankle planter flexors; PFT, ankle plantar flexion torque; TQ, maximal voluntary isometric joint torque.

## Discussion

The main finding obtained here was that isometric maximal joint torques relative to muscle volume in the knee extensor and ankle plantar flexor muscles were not different between the prepubescent and pubescent groups when chronological age was adjusted. This indicates that maturation has little influence on the muscle quality of lower extremity muscles in adolescent boys.

There were significant differences between the pubescent and prepubescent boys in all measured variables except for KET/MV and PFT/MV. The height in the prepubescent boys was small compared to that at peak height velocity of Japanese boys (approximately 154 cm) [28-30]. During puberty, body size changes markedly with advancing chronological age and maturation, and its change accompanies an increase in muscle size and strength [23]. In this study, the subjects were sampled within a limited age range in order to reduce the confounding factor of chronological age. In general, more mature boys are taller and heavier than less mature boys. Thus, the current results reflect the characteristics of normal growth for adolescent boys.

Regardless of the prepubescent and pubescent groups, no significant maturity-related difference was found in the slopes and y-intercepts of the regression lines in the TQ-MV relationships in either muscle, indicating that the TQ-MV relationship in each muscle was similar between the prepubescent and pubescent boys. This is consistent with the earlier findings on the strength-size relationships in upper limb [10,11] and the knee extensors [12,19], but not with that in the gastrocnemius muscle [13]. The discrepancy in the result on the plantar flexors might be attributed to the difference in the subjects examined: prepubescent versus pubescent boys in this study and prepubescent boys versus adults in the earlier study [13]. Furthermore, TQ/MV in both muscles was independent of the Tanner stage (Table 2). Tanner stage is associated with testosterone level [33]. Serum testosterone level is positively related to maximal isokinetic knee extension in adolescent boys [21]. In addition, voluntary activation level during maximal voluntary contraction [37,38], and proportion of fast type fiber [39] have been shown to be higher with increasing age. These findings will support the assumption that muscle quality might be higher in pubescent than in prepubescent boys, as hypothesized at the start of this study. However, this assumption is canceled by the current result that there was no significant difference between the prepubertal and pubertal groups in TQ/MV, with only a trivial or small effect size being observed.

We should comment on methodological issues with the estimation of muscle volume. The muscle volume was estimated using the prediction equation reported by Miyatani *et al*. [35], which has been derived from adult population. It has been shown that pennation angle, fascicle length relative to muscle length, and ratio of synergist muscle to total muscle volume is not different between prepubescent children and adults in the knee extensors [40] and ankle plantar flexors [13]. This implies that in an age span from childhood to adulthood, growth change in muscle volume is not associated with fascicle arrangement. In other words, the muscle thickness and limb length of children will be considered to be a scaled-down geometry of those in adults, and so the prediction equation derived from adult population can be used to estimate muscle volume for children. However, Midorikawa *et al*. [41] reported that while the muscle thickness-based prediction equation for adults are useful for estimating total and regional skeletal muscle mass for adolescents (Tanner stage ≥ II), this equation underestimates muscle mass in prepubescent children. If the previous finding can be applied to our data, the TQ/MV might be higher in the prepubertal group than in the pubertal group due to the underestimation of MV in the prepubertal group. However, the current result refutes this.

In the knee extensors, on the other hand, the y-intercept of the regression line in the relationship between TQ and MV was significantly different from zero, but not for the ankle plantar flexors. This indicates that the higher y-intercept of the regression line from zero appears to result in the overestimation of the TQ/MV in the knee extensors in both groups. It is unknown whether this is due to the use of the prediction equation derived from an adult population. However, it should be noted that the significant difference in the y-intercept of the regression line from zero was found in both prepubertal and pubertal groups. In addition, the y-intercepts and slopes of regression lines in the corresponding relationships were similar between the two groups. These results imply that even if TQ/MV for the knee extensors might be overestimated, its magnitude would be almost the same in both groups and have less influence on the current result that maturity-related difference was not found in KET/MV. To generalize the current results, however, further investigation involving muscle volume measurements with more sophisticated techniques such as magnetic resonance imaging is needed.

## Conclusion

The current results demonstrated that muscle quality, expressed as maximal joint torque relative to muscle volume, is not different between prepubescent and pubescent boys in the knee extensors and ankle plantar flexors. It suggests that maturation has little influence on the strength-size relationship in the lower extremity muscles around puberty. The current results were obtained from a cross-sectional survey. In a longitudinal survey, TQ/MV in the knee extensors is constant for the 6-month period of preadolescence [19]. However, to the best of our knowledge, less information on the longitudinal change in TQ/MV in the period of adolescence is available from earlier findings. To clarify, this is important for pediatric exercise physiology, and further investigation based on longitudinal survey is needed.

## Abbreviations

BMI: Body mass index; KE: Knee extensors; KET: Knee extension torque; L: Limb length; LBM: Lean body mass; MT: Muscle thickness; MV: Muscle volume; MVC: Maximal voluntary isometric joint torque; PCSA: Physiological cross-sectional area; PF: Ankle plantar flexors; PFT: Ankle plantar flexion torque; PH: Stage of pubic hair; TQ: Maximal joint torque; % fat: Percent body fat.

## Competing interests

The authors declare that they have no competing interests.

## Authors’ contributions

YF carried out the anthropometric measurement, performed the statistical analysis and drafted the manuscript. YT conceived of the study, and participated in its design and coordination and helped to draft the manuscript. TY and EF carried out the strength measurement and helped to perform the statistical analysis. MY and HK supervised the survey, participated in the design of the study and performed the statistical analysis. All authors read and approved the final manuscript. All authors read and approved the final manuscript.
